# Pelvic congestion syndrome analysis through quantitative 2-dimensional phase-contrast MRI: a promising vision from an observational cohort study

**DOI:** 10.1097/JS9.0000000000001810

**Published:** 2024-08-02

**Authors:** Chen-Yu Li, Chien-Wei Chen, Chih-Chen Kao, Yin-Chen Hsu, Chung-Yuan Lee, Chieh-Chao Lin, Teng-Yao Yang, Shih-Chung Wang, Sheng-Ya Chen, Yu-Hui Lin, Min Yi Wong, Chee-Jen Chang, Yao-Kuang Huang

**Affiliations:** a Department of Finance, National Taichung University of Science and Technology, Taichung; b Department of Diagnostic Radiology, ChiaYi Chang Gung Memorial Hospital, Chiayi; c College of Medicine, Chang Gung University, Taoyuan; d Division of Thoracic and Cardiovascular Surgery, ChiaYi Chang Gung Memorial Hospital, Chiayi; e Obstetrics and Gynecology, ChiaYi Chang Gung Memorial Hospital, Chiayi; f Cardiology, ChiaYi Chang Gung Memorial Hospital, Chiayi; g Division of Thoracic and Cardiovascular Surgery, Chiayi Hospital, MOHW, Chiayi; h College of Photonics, National Yang Ming Chiao Tung University, Tainan City; i Department of Artificial Intelligence, GICMS and Biomedical Science Research Services Center /Health Information, Chang Gung University, Taoyuan; j Department of Nursing, Chang Gung University of Science and Technology, Chiayi, Taiwan

**Keywords:** congestion, leak, MRI, noncontrast, pelvic, PeVD, phase contrast, venous disease

## Abstract

**Background:**

To examine the application of quantitative 2-dimensional phase-contrast MRI (2D PC-MRI) for treating patients with pelvic congestion syndrome (PCS).

**Materials and Methods:**

The authors conducted a retrospective cross-sectional analysis by using quantitative 2D PC-MRI data enrolled between April 2017 and September 2023. In addition, 32 healthy female controls (HCs) were included.

**Results:**

Most patients with PCS presented with chronic pelvic pain and more than half had extrapelvic venous symptoms (80/81, 98% and 45/81, 56%, respectively). Quantitative 2D PC-MRI analyzed the 81 patients with PCS, 239 patients without PCS, and 32 HCs. The patients with PCS had higher stroke volume (SV), absolute SV (ASV), and mean flux (MF) in the calf region (interstitial pixel shift) than did the HCs. In the left gonadal vein, the patients with PCS had higher SV, backward flow volume (BFV), ASV, and MF and lower forward flow volume (FFV), stroke distance (SD), and mean velocity (MV) than did the HCs. However, the patients with PCS had lower SV, FFV, MF, SD, and MV in the great saphenous veins. Quantitative 2D PC-MRI analysis revealed that the PCS group had higher SV, FFV, BFV, ASV, and MF in the calf region than did the non-PCS group. The variables that most strongly differentiated the patients with PCS from the HCs were SV in the great saphenous veins, SD in the great saphenous veins and left gonadal vein, and MV in the great saphenous veins and left gonadal vein. Caudal flow in the left gonadal vein was identified in half of the patients with PCS (39/81, 48.1%); 14 of them received embolization for left gonadal vein.

**Conclusions:**

In additional to providing an objective 3-dimensional morphology of the pelvic veins and extrapelvic leaks, quantitative 2D PC-MRI analysis reveals distinct hemodynamic profiles between patients with PCS, those without PCS, and HCs, especially in the gonadal veins and regional perfusion of the calves.

## Introduction

Highlights

- Pelvic congestion syndrome (PCS) has a harmless and objective diagnostic tool with hemodynamic information for the venous system. It is called ‘2-dimensional phase-contrast MRI’ (2D PC-MRI).
- Patients with PCS exhibit different QFlow patterns compared to those without PCS or healthy controls. Specifically, there are more negative values (i.e. reversed flow) in the left gonadal vein of patients with PCS compared to others, and this finding is highly correlated with angiography of the left gonadal vein.
- This new technique allows for the identification of detailed pelvic leaks and extrapelvic varices, which are crucial for disease classification and vascular intervention.

Pelvic congestion syndrome (PCS) and pelvic venous disorders are often characterized by vague pain that particularly occurs after prolonged standing, and they occasionally present as dyspareunia with prolonged postcoital aching^1^. The pain is continual and not associated with the menstrual cycle. About 15% of women aged between 18 and 50 years’ experience a pelvic venous disorder^2^. Furthermore, 30–45% of women with chronic pelvic pain (CPP) may have symptoms related to venous disorders^3–5^. PCS is associated with a spectrum of symptoms arising from both reflux, most commonly involving the gonadal and internal iliac veins, and obstruction, often affecting the left renal and iliac veins. These two mechanisms can lead to various conditions, including (a) left flank or abdominal pain and hematuria, (b) CPP, (c) venous claudication, and (d) symptomatic lower extremity varicosities in atypical locations (the vulva or testicles, medial and posterior thigh, and sciatic nerve) or in the typical saphenous distribution, which often recur after initial treatment^6,7^.

Ultrasonography (US), including the transabdominal, intravenous, and transvaginal approaches, is the most widely used noninvasive modality for detecting morphological and hemodynamic abnormalities associated with PCS^8,9^. However, the clinical utility of US is often limited by body habitus and bowel gas, which can prevent the visualization of iliac veins and the inferior vena cava^10^. Moreover, US visualization is subjective, and its hemodynamic assessment can vary between operators. Thus, additional evaluation with cross-sectional imaging modalities, such as contrast-enhanced magnetic resonance angiography (CE-MRA) and computed tomography venogram (CTV), is often required to rule out pelvic pathologies. Although CE-MRA and CTV offer more objective diagnoses and are more suitable for examining pelvic vessels and the inferior vena cava, they provide only morphological insights and no hemodynamic information^11^. Although time-resolved magnetic resonance angiography enables visualization of the dynamic flow of contrast agents, it does not offer quantitative measurements^12,13^.

Noncontrast magnetic resonance angiography (NC-MRA), which incorporates various contrast mechanisms, can be used to evaluate PCS. We have employed NC-MRA with a gated 3-dimensional (3D) turbo spin-echo short tau inversion recovery (TSE-STIR) sequence since 2017 for the diagnosis of lower-extremity venous disorders. In a preliminary study, our technique was determined to be effective when used in combination with US^14,15^. The entire venous anatomy of the lower extremities, particularly the low-flow superficial venous system and pelvic collaterals, can be clearly observed through 3D imaging without the use of a contrast medium or radiation^16,17^. In addition to morphological evaluation using the gated 3D TSE-STIR sequence, we conduct quantitative analysis by using a 2-dimensional (2D) phase-contrast (PC) MRI sequence for hemodynamic measurements of various venous segments. Currently, quantitative PC-MRI is being used to examine the cerebrospinal fluid, cardiovascular system, and aortic diseases^18–20^. However, this technique has rarely been employed in the diagnosis of pelvic vascular diseases. At our institution, an MRI protocol combining gated 3D TSE-STIR MRV with quantitative 2D PC-MRI has become the standard preoperative assessment for lower-extremity venous disease and has proven effective^16,17,21–27^. In this study, we analyzed and compared the quantitative 2D PC-MRI data of patients with PCS, those without PCS, and healthy volunteers (healthy controls, HCs).

## Materials and methods

### Participants

**Figure 1:**
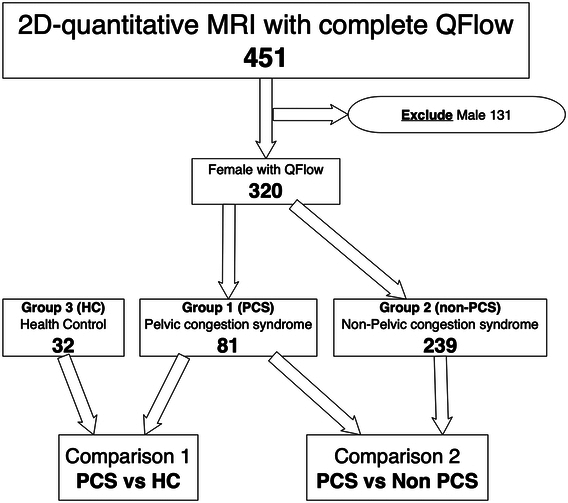
Flow chart of the study cohort.

This is an observational cohort study conducted at a single tertiary medical center. We collected comprehensive data from 405 individuals who visited our outpatient department for suspected pelvic and lower extremity venous disorders. The participants underwent NC-MRA combined quantitative PC-MRI between April 2017 and September 2023. All data were prospectively collected and retrospectively analyzed. We excluded patients who were pregnant, had restless leg syndrome, had arrhythmia, were morbidly obese, and had devices incompatible with MRI. Initially, the 131 male patients were excluded. The remaining 320 female patients were divided into two groups. The first group consisted of 81 females with pelvic venous congestion syndrome (PCS) of pain dominant presentation^28^. During the examination, ultrasonography was performed in a supine position. The criteria for diagnosing PCS via ultrasound involved detecting pelvic varicose veins indicating the presence of tortuous and dilated veins measuring 5 mm or more in diameter around the ovary and uterus. Before their scheduled MRI, patients diagnosed with PCS underwent a gynecological evaluation to rule out potential malignancies. For this study, the third group consisted of 32 healthy volunteers who were designated as controls. These healthy controls (HCs) were adult women with no known vascular disease in their lower extremities. To evaluate their venous condition and collect data, they underwent an MRI. All MRI images were interpreted by a radiologist (CWC) with 10 years of experience in vascular radiology. Please refer to Figure 1 for a flowchart of the patient identification and exclusion for the study cohort.

The 81 patients with PCS had relevant symptoms and signs, including pelvic venous pain, dyspareunia, discomfort or heaviness in the hypogastric region, and vulvar varicose veins. Quantitative 2D PC-MRI data confirmed reflux in the left gonadal vein, parametrial varicose veins, and uterine varicose veins, with typical morphology. In addition, we recorded information on the patients’ demographics, visual analog scale (VAS) scoring, Symptoms–Varices–Pathophysiology (SVP) classification, venous characteristics, medications, and interventions for venous diseases^28^. The visual analog scale (VAS) is a straightforward tool used to measure pain intensity. It consists of a straight line labeled with ‘no pain’ and ‘worst imaginable pain’ at the endpoints. Patients can indicate the level of pain they are experiencing by making a mark on the line. This study utilizes a simplified VAS method that provides a pain score ranging from 0 to 10 to quantify the intensity of the pain. The Symptoms-Varices-Pathophysiology (SVP) classification is a comprehensive tool that has been developed to classify pelvic venous disorders. This system is comprised of three main domains: Symptoms (S), which documents the patient’s clinical signs; Varices (V), which identifies the involved varicose veins; and Pathophysiology (P), which is subdivided into Anatomic (A), Hemodynamic (H), and Etiologic (E) aspects. This classification system is useful for improving the diagnosis, tailoring treatments, and facilitating research by defining homogeneous patient groups. This work was reported according to the Strengthening the Reporting of Cohort Studies in Surgery (STROCSS, Supplemental Digital Content 1, http://links.lww.com/JS9/D237) criteria^29^.

### MRI data acquisition

All MRI data were collected using a 1.5-T MRI scanner (Philips Ingenia, Philips Healthcare, Best). Technicians attached electrocardiography electrodes to the patient’s chest to monitor their heart rhythm and then used a heart synchronization method to obtain images. The MRI protocol included gated 3D TSE-STIR MRV for anatomical diagnosis and quantitative 2D PC-MRI for hemodynamic analysis. In the TSE sequence, arterial blood flow is rapid, leading to flow voids during systole. Thus, when the 3D TSE-STIR sequence is used to trigger image acquisition during systole, the resulting 3D dataset features only venous structures because STIR additionally suppresses background signals from fat and bone. Three-dimensional TSE-STIR MRV images were acquired at four coronal plane levels (the abdomen, pelvis, thigh, and lower leg) by using the following parameters: repetition time, 1 beat; echo time, 85 ms; inversion recovery delay time, 160 ms; voxel size, 1.7 mm×1.7 mm×4 mm; and field of view, 360 mm×320 mm. Quantitative 2D PC-MRI was performed at four axial planes by using the following parameters: repetition time, 16 ms; echo time, 8 ms; tilt angle, 10°; 25 images/period; slice thickness, 5 mm; pixel size, 0.33 mm×0.33 mm; and velocity encoding, 80 cm/s. The entire MRI process—including image acquisition, image reconstruction, and 2D PC-MRI analysis—required 25 min.

### Hemodynamic variables

**Figure 2:**
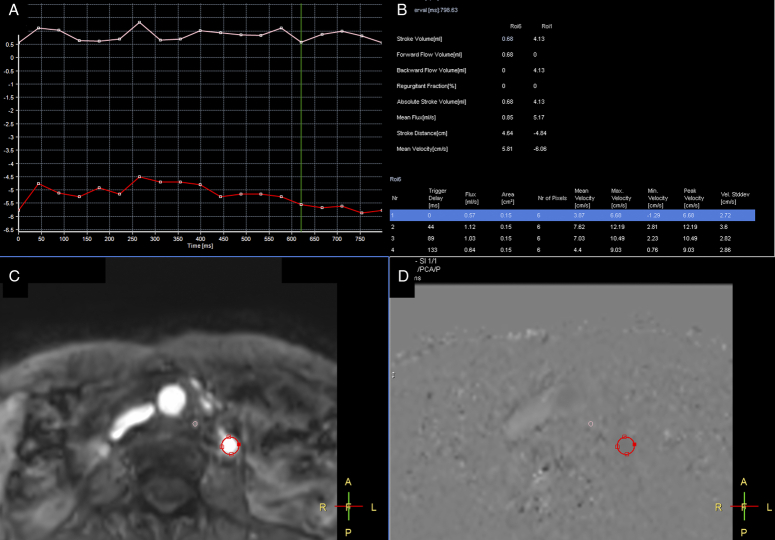
QFlow process for left gonadal vein. A, Recording phase shift of the pixels by time: left gonadal vein is indicated by red-line. B, QFlow parameters record by different region of interest (ROI). C, The red circle indicates the left gonadal vein as ROI. D, Processing QFlow analysis for ROI.

**Figure 3:**
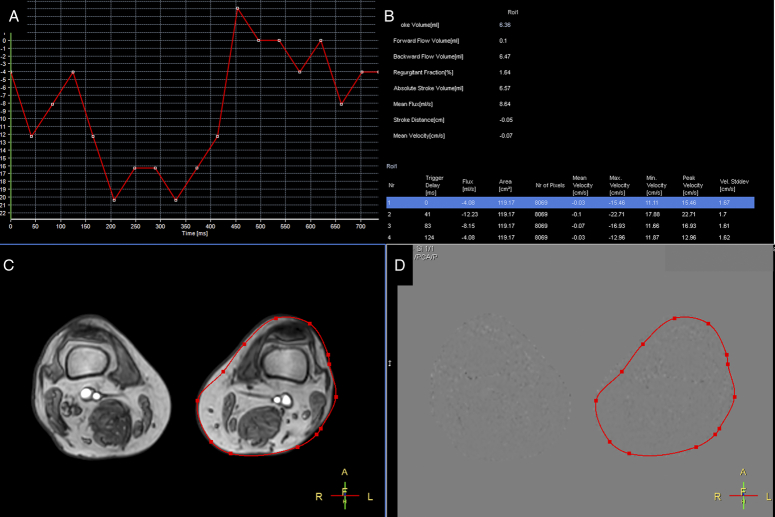
QFlow process for left calf region (interstitial pixel shift). A. Recording phase shift of the pixels by time: the vascular signal has been removed. B. QFlow parameters record by region of interest (ROI). C, The red circle indicates the whole left calf as ROI. D, Processing QFlow analysis for ROI.

An experienced technician (SCW) delineated the region of interest (ROI) on the vascular lumens, covering the whole lumen at axial planes of the inferior vena cava, external iliac vein, femoral vein, popliteal vein, great saphenous vein, and left gonadal vein (Fig. 2). Then, variables were generated by measuring the phase-shifting information of voxels within the ROI. In addition, by drawing the ROI to include the entire cross-section of the calves and filtering out vessel signals and background noise, we created images representing the right and left regional calves (RG and LG), indicating the pixel shift in the interstitial tissue of the calves.(Fig. 3) The following seven variables were calculated: the stroke volume (SV), calculated as the net blood volume passing through the lumen during one heartbeat; forward flow volume (FFV), calculated as the blood volume passing through the lumen in the positive direction (toward the head) during one heartbeat; backward flow volume (BFV), calculated as the blood volume passing through the lumen in the negative direction (toward the foot) during one heartbeat; absolute stroke volume (ASV), calculated as the sum of the absolute values of the FFV and BFV; mean flux (MF), calculated as stroke volume × heartbeat/[60 × (1 − heartbeat)]; stroke distance (SD), calculated as the net distance that blood travels in the vessel during one heartbeat; and mean velocity (MV), calculated as stroke distance × heartbeat/[60 × (1 − heartbeat)].

### Statistical analysis

The demographic characteristics of the patients were compared using one-way analysis of variance for continuous variables and Fisher’s exact test for categorical variables. In accordance with the primary diagnosis established using US, we compared the clinical characteristics of the patients with venous reflux with those of the patients with venous obstruction by using the independent-sample *t*-test for age and Fisher’s exact test for categorical variables. Cohen’s Kappa coefficient (k) was calculated to evaluate interrater reliability between the venogram and 2D PC MRI in the left gonadal vein. Because the data are non-normally distributed, the 2D PC-MRI data are presented as the median and interquartile range. The 2D PC-MRI data for the patients with pelvic venous disorders and for the HCs were compared using the Kruskal–Wallis test, with a Bonferroni adjustment for post-hoc comparisons. The ability of 2D PC-MRI to differentiate the diagnoses (i.e. PCS from HC and PCS from no PCS) was determined through receiver operating characteristic curve analyses. The cutoff was determined using the Youden index. All tests were 2-tailed, and *P*<0.05 was considered statistically significant. Data analyses were conducted using SPSS 25 (IBM SPSS Inc.).

## Results

### Participant characteristics

Table 1 lists the clinical information of the all subjects by groups (PCS, non-PCS, and HC), including age, comorbidities, relevant symptoms and signs (SVP classification), venous morphology on MRI, visual analog scale (VAS) scores, medication for venous disorders, and venous intervention. Most of the patients experienced chronic pelvic pain, with some reporting extrapelvic symptoms, such as dyspareunia, genital discomfort, nonsaphenous thigh pain, sciatica, and venous claudication. Venous morphology was predominantly characterized by pelvic varices, including some varicose veins over the genital area and pelvic escape points (Video, Supplemental Digital Content 2, http://links.lww.com/JS9/D238). Notably, 39 of the 81 patients with PCS (48.1%) exhibited caudal flow in the left gonadal vein and 42 (51.8%) had a bigger left gonadal vein, as confirmed by quantitative 2D PC-MRI. More than half of the patients with PCS received micronized purified flavonoid fraction, pentoxifylline, and nonsteroidal anti-inflammatory drugs. Furthermore, 14 patients received embolization for the left gonadal vein, 19 patients received great saphenous vein truncal ablation plus sclerotherapy, and 1 patient received angioplasty for her left external iliac vein. One patient with pelvic congestion syndrome underwent left external iliac vein angioplasty because of the comorbidity of iliac vein compression syndrome.

**Table 1:** Clnical information of patients with pelvic congestion syndrome (PCS), patients with nonpelvic congestion syndrome venous disease (non-PCS), and health controls (HC).

	PCS (*N*=81)	Non-PCS (*N*=239)	HC (*N*=32)
Age (years±SD)	55.44±14.52	64±14.51	34.56±14.03
Comorbidities			
Smoking	2	2	0
Hypertension	21	74	0
Diabetes	8	38	0
Cerebral vascular accident (Stoke)	1	1	0
Coronary arterial disease	2	5	0
History of malignancy	2	33	0
Clinical manifestation			
Pelvic congesting syndrome (Pain/itch/dullness)			
Back pain (SVP, S1)	7	3	0
Chronic pelvic pain (SVP S2)	80	2	0
Extrapelvic symptoms of venous origin (SVP S3)	45	10	0
Dyspareunia, genital pain, ovarian tenderness (SVP S3a)	14	2	0
Posteriomedial thigh (nonsaphenous) pain and Sciatica (SVP S3b)	16	3	0
Venous claudication (SVP S3c)	26	22	0
VAS	3.99±1.33	1.17±0.98	0
Unhealed foot wound	0	23	0
Asymmetric Swollen legs	32	76	0
Discolor, soreness or cosmatic issue of leg	20	123	0
Vague lower body discomfort	0	17	0
Pelvic venous morphology			
Renal hilar varices (SVP V1)	1	7	0
Pelvic varices (SVP V2)	79	14	2
Pelvic origin extrapelvic varices (SVP V3)	33	12	0
Genital varices (SVP V3a)	25	10	0
Pelvic origin varicose vein from pelvic escape points (SVP V3b)	20	2	0
Pathophysiology			
Obstruction	11	87	0
Reflux	70	98	0
Nonspecific	0	54	32
Etiology			
Non thrombotic	81	190	0
Thrombotic	0	49	0
MRI figures for veins			
LGV diameter over 4 mm	63	12	3
LGV diameter over 8 mm	42	9	0
LGV caudal flow	39	6	0
Uterine varices over 5 mm	66	30	2
Vulva and genital varices	39	21	0
GSV varices	24	33	0
Lateral thigh varices	31	63	0
Medication			
Micronized purified flavonoid fraction	41	20	0
Pentoxifylline	53	137	0
Conazepam	12	35	0
NSAID	45	98	0
Intervention			
LGV embolization	14	0	0
Sclerotherapy (Fibrovein)	15	62	0
GSV ablation	19	73	0
Angioplasty	1	7	0
Stenting in iliac veins	0	14	0

GSV, Greater saphenous vein; LGV, left gonadal vein; SVP, Symptoms-Varices-Pathophysiology classification; VAS, visual analog scale.

### Quantitative 2D PC-MRI (QFlow) analysis of PCS and control groups

Table 2 lists the findings of quantitative 2D PC-MRI hemodynamic analysis for the 81 patients with PCS, the 32 HCs and 239 patients with non-PCS. We recorded the SV, MF, SD, and MV in the inferior vena cava, external iliac vein, femoral vein, great saphenous vein, popliteal vein, left gonadal vein (left ovarian vein), and calf region (interstitial pixel shift). 2D PC-MRI analysis revealed significant differences in these parameters between the patients with PCS, the HCs. In particular, the patients with PCS had lower SV in the great saphenous veins (right great saphenous vein, *P*=0.0125; left great saphenous vein, *P*=0.0024), higher SV in the left gonadal vein (*P*=0.001), higher SV in the calf region (RG, *P*=0.0071; LG, *P*=0.0151), lower FFV in the great saphenous veins (right great saphenous vein, *P*=0.0134; left great saphenous vein, *P*=0.0028), lower FFV in the left gonadal vein (*P*=0.0135), and higher FFV in the right calf region (RG, *P*=0.0253). Moreover, these patients had higher BFV in the left great saphenous vein (*P*=0.0213), left gonadal vein (*P*=0.0001), and left calf region (LG, *P*=0.0124); higher ASV in the left gonadal vein (*P*=0.0001), RG (*P*=0.0049), and LG (*P*=0.0078); lower MF in the great saphenous veins (right great saphenous vein, *P*=0.0126; left great saphenous vein, *P*=0.0094); higher MF in the right external iliac vein (*P*=0.0412), left gonadal vein (*P*=0.0002), and calf region (RG, *P*=0.0127; LG, *P*=0.0228); smaller SD in the great saphenous veins (*P*=0.0001) and left gonadal vein (*P*=0.0001); greater SD in the left femoral vein (*P*=0.0489); and lower MV in the great saphenous veins (*P*=0.0001) and the left gonadal vein (*P*=0.0001).

**Table 2:** Patients with pelvic congestion syndrome (PCS), health control, and non-PCS: QFlow parameter in different venous segments and calves’ regions.

ROI	Pelvic congestion syndrome (PCS, *n*=81)	Health control (HC, *n*=32)	Nonpelvic congestion syndrome (Non-PCS, *n*=239)
Stroke Volume (SV), ml			
IVC	15.633 (14.505–16.762)	15.734 (13.482–17.986)	13.329** (12.433–14.224)
REIV	4.6004 (4.1754–5.0253)	3.9838 (3.4202–4.5473)	4.9349 (4.6148–5.2550)
RFV	1.2489 (1.1085–1.3893)	1.2800 (1.0657–1.4943)	1.4498* (1.3435–1.5561)
RGSV	0.2380 (0.1594–0.3166)	0.3869* (0.2994–0.4743)	0.2702 (0.2268–0.3136)
LGSV	0.2268 (0.1536–0.2999)	0.4153** (0.3193–0.5114)	0.3134 (0.2463–0.3805)
RPV	0.7110 (0.6057–0.8163)	0.5944 (0.4856–0.7032)	0.8863* (0.8027–0.9699)
LPV	0.6878 (0.5968–0.7787)	0.5744 (0.4715–0.6773)	0.8804** (0.7855–0.9752)
LGV	1.2575 (0.9459–1.5692)	0.5619*** (0.4053–0.7185)	0.5397*** (0.3391–0.7404)
RG	0.3141 (0.1756–0.4526)	0.1084** (0.0523–0.1646)	0.1120** (0.0846–0.1393)
LG	0.3331 (0.2092–0.4569)	0.1519* (0.0724–0.2314)	0.1058*** (0.0756–0.1360)
Forward flow volume (FFV), ml			
IVC	16.068 (14.915–17.221)	16.092 (13.832–18.353)	13.654** (12.742–14.566)
RFV	1.2559 (1.1159–1.3959)	1.2959 (1.0830–1.5088)	1.4588* (1.3528–1.5647)
RGSV	0.2442 (0.1652–0.3232)	0.3903* (0.3045–0.4761)	0.2777 (0.2346–0.3208)
LGSV	0.2325 (0.1599–0.3051)	0.4166** (0.3210–0.5121)	0.3037 (0.2490–0.3584)
RPV	1.2507 (1.0997–1.4018)	0.6059 (0.4995–0.7124)	1.4834* (1.3635–1.6034)
LPV	0.6893 (0.5985–0.7800)	0.5781 (0.4773–0.6789)	0.8772** (0.7815–0.9728)
LGV	0.2867 (0.2001–0.3733)	0.5247* (0.3573–0.6921)	0.2888 (0.1301–0.4476)
RG	0.2563 (0.1152–0.3974)	0.0850* (0.0328–0.1372)	0.0913* (0.0667–0.1159)
LG	0.2884 (0.1596–0.4172)	0.1422* (0.0629–0.2215)	0.1011** (0.0723–0.1299)
Backward flow volume (BFV), ml			
RGSV	0.0086 (0.0035–0.0137)	0.0040 (−0.0012 to 0.0093)	0.0168* (0.0119–0.0217)
LGSV	0.0072 (0.0024–0.0121)	0.0012* (−0.0005 to 0.0030)	0.0392 (−0.0085 to 0.0870)
RPV	0.0063 (0.0011–0.0114)	0.0103 (0.0020–0.0186)	0.0217* (0.0090–0.0344)
LPV	0.0023 (0.0003–0.0043)	0.0037 (−0.0015 to 0.0090)	0.0228 (0.0010–0.0445)
LGV	1.0225 (0.6850–1.3600)	0.0597*** (0.0138–0.1056)	0.2888*** (0.1572–0.4205)
RG	0.0986 (0.0661–0.1312)	0.0541 (0.0155–0.0926)	0.0294*** (0.0168–0.0420)
LG	0.0899 (0.0548–0.1249)	0.0316* (0.0016–0.0615)	0.0210** (0.0090–0.0330)
Absolute stroke volume (ASV), ml			
LGV	1.3110 (1.0044–1.6176)	0.5856*** (0.4368–0.7345)	0.5787*** (0.3786–0.7787)
RG	0.3585 (0.2206–0.4965)	0.1409** (0.0795–0.2024)	0.1260** (0.0978–0.1543)
LG	0.3814 (0.2522–0.5105)	0.1753** (0.0946–0.2560)	0.1231*** (0.0922–0.1540)
Mean flux (MF), ml/s			
IVC	18.119 (16.771–19.466)	18.417 (15.663–21.170)	15.602** (14.505–16.700)
REIV	5.2112 (4.7032–5.7193)	4.3688* (3.7268–5.0107)	5.6517 (5.2688–6.0346)
RFV	1.4188 (1.2493–1.5882)	1.4072 (1.1455–1.6689)	1.6587* (1.5335–1.7839)
LFV	1.4059 (1.2200–1.5918)	1.3337 (1.0424–1.6251)	1.6938* (1.5562–1.8314)
RGSV	0.2630 (0.1775–0.3484)	0.4309* (0.3294–0.5325)	0.3101 (0.2586–0.3616)
LGSV	0.2656 (0.1748–0.3563)	0.4516** (0.3436–0.5595)	0.3498 (0.2805–0.4192)
RPV	0.8046 (0.6743–0.9348)	0.6513 (0.5250–0.7775)	1.0165* (0.9192–1.1139)
LPV	0.7754 (0.6658–0.8851)	0.6334 (0.5116–0.7553)	1.0037** (0.8962–1.1111)
LGV	1.4883 (1.0940–1.8825)	0.6437*** (0.4637–0.8238)	0.6209*** (0.4079–0.8339)
RG	0.3525 (0.1880–0.5170)	0.1269* (0.0601–0.1936)	0.1255** (0.0951–0.1559)
LG	0.3669 (0.2267–0.5071)	0.1744* (0.0833–0.2654)	0.1213** (0.0865–0.1562)
Stroke distance (SD), cm			
IVC	9.9285 (9.0915–10.765)	10.647 (8.7638–12.531)	8.1826** (7.5630–8.8023)
RFV	2.7875 (2.4471–3.1280)	3.7409 (2.8399–4.6420)	3.1533* (2.9196–3.3870)
LFV	2.9541 (2.6202–3.2879)	4.1309* (3.0033–5.2586)	3.3484 (3.0722–3.6245)
RGSV	1.0363 (0.7536–1.3190)	2.1647*** (1.7050–2.6243)	1.2356 (1.0409–1.4303)
LGSV	1.0006 (0.7365–1.2648)	2.4613*** (1.9707–2.9518)	1.3609 (1.0839–1.6379)
RPV	1.2152 (1.0601–1.3702)	1.0678 (0.9175–1.2182)	1.5104** (1.3728–1.6480)
LGV	−0.5905 (−1.3923 to 0.2114)	2.2578*** (1.3450–3.1707)	0.2578 (−0.1047–0.6202)
Mean velocity (MV), cm/s			
IVC	11.549 (10.582–12.517)	12.481 (10.358–14.605)	9.4973*** (8.7949–10.199)
RFV	3.1667 (2.7490–3.5843)	4.1297 (3.0628–5.1966)	3.6184 (3.3381–3.8987)
RGSV	1.1614 (0.8424–1.4803)	2.3678*** (1.8603–2.8754)	1.3979 (1.1767–1.6192)
LGSV	1.1606 (0.8397–1.4815)	2.6694*** (2.1147–3.2240)	1.5621 (1.2558–1.8684)
RPV	1.3774 (1.1877–1.5671)	1.1653 (0.9882–1.3424)	1.7385** (1.5764–1.9007)
LGV	−0.7837 (−1.7147 to 0.1473)	2.5581*** (1.5061–3.6102)	0.2673* (−0.1530 to 0.6876)

* *P*<0.05,

** *P*<0.01,

*** *P*<0.0001.

IVC, inferior vena cava; LEIV, left external iliac vein; LFV, left femoral vein; LG, left calf region; LGSV, left great saphenous vein; LGV, left gonadal vein (left ovarian vein); LPV, left popliteal vein; REIV, right external iliac vein; RFV, right femoral vein; RG, right calf region; RGSV, right great saphenous vein; ROI: region of interest; RPV, right popliteal vein.

The variables that most strongly differentiated the female patients with PCS from the HCs were the SV in the great saphenous veins (right great saphenous vein, area under the curve [AUC]=73%, 95% CI=64–82%; left great saphenous vein, AUC=75%, 95% CI=66–84%), SV in the left gonadal vein (AUC=63%, 95% CI=53–74%), SV in the right calf region (AUC=70%, 95% CI=59–80%), SV in the left calf region (AUC=66%, 95% CI=55–78%), MF in the right external iliac vein (AUC=62%, 95% CI=51–73%), MF in the great saphenous veins (right great saphenous vein, AUC=72%, 95% CI=63–82%; left great saphenous vein, AUC=74%, 95% CI=66–83%), MF in the left gonadal vein (AUC=64%, 95% CI=59–80%), MF in the right calf region (AUC=69%, 95% CI=59–80%), MF in the left calf region (AUC=66%, 95% CI=54–78%), SD in the great saphenous veins (right great saphenous vein, AUC=75%, 95% CI=66–85%; left great saphenous vein, AUC=79%, 95% CI=71–88%), SD in the left gonadal vein (AUC=72%, 95% CI=62–82%), MV in the great saphenous veins (right great saphenous vein, AUC=75%, 95% CI=65–84%; left great saphenous vein, AUC=78%, 95% CI=69–86%), and MV in the left gonadal vein (AUC=72%, 95% CI=62–82%). The patients with PCS had more negative values in SD and MV of the left gonadal vein than did the HCs (*P*<0.0001; Table 3).

**Table 3:** ROC test for pelvic congestion syndrome (*n*=81) and health controls (*n*=32).

Parameters	OR (95% CI)	*P*	AUC (95% CI)	*P* *
Stroke volume (SV), ml				
REIV	1.208 (0.957–1.526)	0.1116	0.6055 (0.4914–0.7196)	0.0698
RGSV	0.283 (0.083–0.961)	0.0429	0.7309 (0.6387–0.8231)	<0.0001*
LGSV	0.179 (0.049–0.654)	0.0093	0.7512 (0.6640–0.8383)	<0.0001*
LGV	2.245 (1.167–4.318)	0.0154	0.6321 (0.5276–0.7367)	0.0132
RG	49.79 (3.396–730.1)	0.0043	0.6991 (0.5939–0.8042)	0.0002*
LG	7.363 (1.008–53.80)	0.0049	0.6644 (0.5500–0.7787)	0.0048*
Mean flux (MF), ml/s				
REIV	1.210 (0.985–1.485)	0.0688	0.6215 (0.5092–0.7339)	0.0340
LEIV	1.168 (0.941–1.450)	0.1579	0.6136 (0.4983–0.7289)	0.0534
RGSV	0.303 (0.097–0.945)	0.0396	0.7242 (0.6309–0.8174)	<0.0001*
LGSV	0.318 (0.112–0.903)	0.0315	0.7442 (0.6561–0.8323)	<0.0001*
LGV	2.004 (1.119–3.588)	0.0194	0.6381 (0.5342–0.7421)	0.0092
RG	26.48 (2.569–272.9)	0.0059	0.6943 (0.5869–0.8016)	0.0004*
LG	5.228 (0.880–31.06)	0.0689	0.6591 (0.5428–0.7754)	0.0073*
Stroke distance (SD), cm				
RFV	0.772 (0.611–0.974)	0.0289	0.6088 (0.4831–0.7345)	0.0897
LFV	0.782 (0.640–0.955)	0.0161	0.5733 (0.4484–0.6982)	0.2500
RGSV	0.540 (0.387–0.753)	0.0003	0.7533 (0.6586–0.8480)	<0.0001*
LGSV	0.447 (0.313–0.639)	<0.0001	0.7934 (0.7117–0.8751)	<0.0001*
LGV	0.750 (0.640–0.879)	0.0004	0.7211 (0.6244–0.8177)	<0.0001
Mean velocity (MV), cm/s				
RFV	0.839 (0.699–1.006)	0.0581	0.5870 (0.4601–0.7138)	0.1789
LFV	0.839 (0.716–0.983)	0.0298	0.5561 (0.4299–0.6823)	0.3833
RGSV	0.588 (0.436–0.794)	0.0005	0.7490 (0.6538–0.8443)	<0.0001*
LGSV	0.552 (0.414–0.736)	<0.0001	0.7784 (0.6947–0.8620)	<0.0001*
LGV	0.772 (0.671–0.889)	0.0003	0.7216 (0.6249–0.8184)	<0.0001*
Negative value (reflux) in left gonadal vein				
SD of LGV	0.750 (0.640–0.879)	0.0004	0.7211 (0.6244–0.8177)	<0.0001*
MV of LGV	0.772 (0.671–0.889)	0.0003	0.7216 (0.6249–0.8184)	<0.0001*

* *P*<0.05

IVC, inferior vena cava; LEIV, left external iliac vein; LFV, left femoral vein; LG, left calf region; LGSV, left great saphenous vein; LGV, left gonadal vein (left ovarian vein); LPV, left popliteal vein; REIV, right external iliac vein; RFV, right femoral vein; RG, right calf region; RGSV, right great saphenous vein; ROI, region of interest; RPV, right popliteal vein.

**Figure 4:**
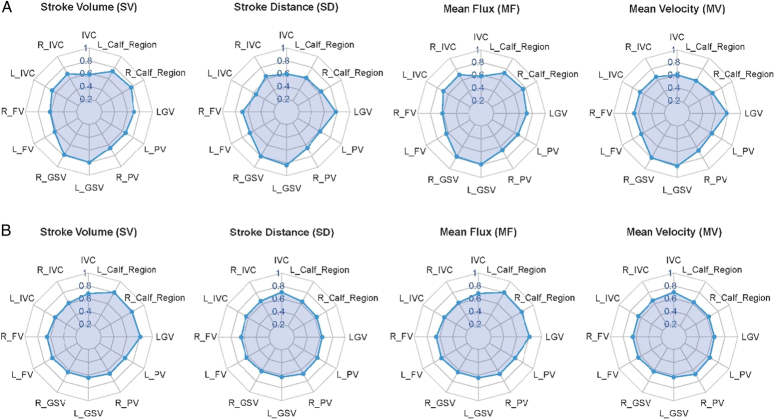
Radar chart of the QFlow parameters in venous segments. a (upper row) It presented the values of AUC (Area Under Curve) of the patients with PCS in SV, SD, MF, and MV based on comparison with the patients with HC in 2D PC-MRI. These radar charts were composed of 12 AUC values, including IVC, left calf ankle, right calf ankle, LGV, left PV, right PV, left GSV, right GSV, left FV, right FV, left IVC, right IVC, respectively. The AUC value ranges between 0 and 1, and a larger AUC indicates better discrimination of PCS relative to HC. The area showed that the area was 1.1903 in MF, 1.1786 in SV, 1.1383 in MV, and 1.1005 in SD, and all 4 plots can discriminate the patients with PCS relative to the HC on the benchmark of distinguishable value. b (lower row) This chart primarily presented the values of AUC (Area Under Curve) of the patients with PCS in SV, SD, MF, and MV based on comparison with the patients with non-PCS in 2D PC-MRI diagnosed approach. These radar charts were composed of 12 AUC values, including IVC, left calf ankle, right calf ankle, LGV, left PV, right PV, left GSV, right GSV, left FV, right FV, left IVC, right IVC, respectively. The AUC value ranges between 0 and 1, and a larger AUC indicates better discrimination of PCS relative to non-PCS. The area was 1.1710 in MF, 1.1650 in SV, 0.9834 in MV, and 0.9782 in SD under the assumption of a unit circle in the radar charts.

The radar chart visualized the discrimination ability of QFlow parameters in each venous segments between patients with PCS and heath control (Fig. 4A).

### 2D PC-MRI QFlow analysis in PCS and non-PCS groups

During the study period, 239 patients presenting with conditions other than PCS (non-PCS) received 2DPC-MRI. The parameters of the 81 patients with PCS also significantly differed from those of the 239 patients without PCS (Table 2). In particular, the patients with PCS had higher SV in the inferior vena cava (*P*=0.0018), lower SV in the femoral veins (right femoral vein, *P*=0.0249; left femoral vein, *P*=0.0151), lower SV in the popliteal veins (right popliteal vein, *P*=0.0105; left popliteal vein, *P*=0.004), higher SV in the left gonadal vein (*P*=0.002), higher SV in the calf region (RG, *P*=0.0055; LG, *P*=0.0006), higher FFV in the inferior vena cava (*P*=0.0018), lower FFV in the femoral veins (right femoral vein, *P*=0.0231; left femoral vein, *P*=0.0178), lower SV in the popliteal veins (right popliteal vein, *P*=0.0086; left popliteal vein, *P*=0.0052), and higher FFV in the calf region (RG, *P*=0.0244; LG, *P*=0.0059). Moreover, these patients had higher BFV in the right great saphenous vein (*P*=0.0228), left gonadal vein (*P*=0.0001), and calf region (RG, *P*=0.0001; LG, *P*=0.0004); higher ASV in the left gonadal vein (*P*=0.0001), RG (*P*=0.0015), and LG (*P*=0.0002); higher MF in the inferior vena cava (*P*=0.0046); lower MF in the femoral veins (right femoral vein, *P*=0.0252; left femoral vein, *P*=0.0145); lower MF in the popliteal veins (right popliteal vein, *P*=0.0106; left popliteal vein, *P*=0.0036); higher MF in the left gonadal vein (*P*=0.0002) and calf region (RG, *P*=0.0084; LG, *P*=0.0011); higher SD in the inferior vena cava (*P*=0.011); lower SD in the right popliteal vein (*P*=0.052); higher MV in the inferior vena cava (*P*=0.0008); lower MV in the right popliteal vein (*P*=0.0046); and lower MV in the left gonadal vein (*P*=0.0432). There are similar trends of those parameters between the patients with PCS and the others (non-PCS and HCs) (Table 4).

**Table 4:** Patients with pelvic congestion syndrome (PCS) and the others (HC and Non-PCS): QFlow parameter in different venous segments and calves’ regions.

ROI	Pelvic congestion syndrome (PCS, *n*=81)	The others (*n*=270)	*P*
Stroke volume (SV), ml			
IVC	15.648 (14.533–16.763)	13.601 (12.764–14.437)	0.0040*
RFV	1.2550 (1.1158–1.3942)	1.4286 (1.3313–1.5258)	0.0443
LFV	1.2462 (1.0960–1.3964)	1.4470 (1.3381–1.5559)	0.0333
LGSV	0.2313 (0.1585–0.3042)	0.3244 (0.2640–0.3849)	0.0526
RPV	0.7194 (0.6141–0.8247)	0.8498 (0.7741–0.9255)	0.0478
LPV	0.6913 (0.6013–0.7814)	0.8437 (0.7582–0.9292)	0.0159
LGV	1.2512 (0.9432–1.5593)	0.5416 (0.3633–0.7200)	0.0001*
RG	0.3102 (0.1732–0.4472)	0.1120 (0.0870–0.1370)	0.0058*
LG	0.3290 (0.2065–0.4516)	0.1116 (0.0834–0.1399)	0.0009*
Forward flow volume (FFV), ml			
IVC	16.077 (14.938–17.216)	13.931 (13.080–14.782)	0.0032*
RFV	1.2620 (1.1232–1.4007)	1.4384 (1.3415–1.5353)	0.0402*
LFV	1.2546 (1.1052–1.4040)	1.4506 (1.3408–1.5603)	0.0376*
LGSV	0.2370 (0.1647–0.3092)	0.3160 (0.2662–0.3657)	0.0759
RPV	0.7261 (0.6219–0.8303)	0.8601 (0.7846–0.9355)	0.0404*
LPV	0.6928 (0.6029–0.7827)	0.8413 (0.7552–0.9275)	0.0190*
RG	0.2532 (0.1137–0.3927)	0.0909 (0.0684–0.1134)	0.0248*
LG	0.2849 (0.1575–0.4123)	0.1064 (0.0793–0.1334)	0.0077*
Backward flow volume (BFV), ml			
REIV	0.0734 (0.0331–0.1137)	0.1330 (0.0768–0.1893)	0.0895
RGSV	0.0085 (0.0035–0.0135)	0.0153 (0.0109–0.0197)	0.0443*
RPV	0.0062 (0.0011–0.0113)	0.0204 (0.0091–0.0317)	0.0239*
LPV	0.0023 (0.0003–0.0042)	0.0206 (0.0013–0.0399)	0.0633
LGV	1.0100 (0.6758–1.3442)	0.2627 (0.1459–0.3796)	0.0001*
RG	0.0974 (0.0651–0.1296)	0.0324 (0.0204–0.0444)	0.0003*
LG	0.0888 (0.0541–0.1235)	0.0224 (0.0112–0.0335)	0.0005*
Absolute stroke volume (ASV), ml			
LGV	1.3040 (1.0009–1.6071)	0.5789 (0.4012–0.7566)	0.0001*
RG	0.3541 (0.2176–0.4906)	0.1283 (0.1023–0.1542)	0.0017*
LG	0.3767 (0.2489–0.5046)	0.1297 (0.1009–0.1586)	0.0003*
Mean flux (MF), ml/s			
IVC	18.132 (16.801–19.463)	15.922 (14.898–16.947)	0.0099*
RFV	1.4249 (1.2571–1.5926)	1.6279 (1.5129–1.7429)	0.0494*
LFV	1.4099 (1.2261–1.5936)	1.6510 (1.5242–1.7778)	0.0337*
RPV	0.8137 (0.6837–0.9436)	0.9713 (0.8829–1.0596)	0.0483*
LPV	0.7793 (0.6707–0.8878)	0.9595 (0.8624–1.0566)	0.0150*
LGV	1.4805 (1.0908–1.8701)	0.6227 (0.4333–0.8122)	0.0001*
RG	0.3482 (0.1855–0.5108)	0.1261 (0.0982–0.1540)	0.0089*
LG	0.3624 (0.2237–0.5012)	0.1281 (0.0955–0.1606)	0.0015*
Stroke distance (SD), cm			
IVC	9.9275 (9.1009–10.754)	8.4686 (7.8732–9.0640)	0.0051*
RFV	2.7920 (2.4556–3.1283)	3.2229 (2.9915–3.4544)	0.0379*
LFV	2.9777 (2.6447–3.3107)	3.4354 (3.1585–3.7123)	0.0375*
RGSV	1.0567 (0.7746–1.3388)	1.3403 (1.1571–1.5234)	0.0965
LGSV	1.0298 (0.7625–1.2970)	1.4838 (1.2294–1.7382)	0.0156*
RPV	1.2250 (1.0706–1.3794)	1.4560 (1.3320–1.5801)	0.0218*
LGV	−0.4898 (−1.3066 to 0.3271)	0.4674 (0.1258–0.8089)	0.0339*
LG	0.0037 (0.0007–0.0066)	0.0011 (0.0005–0.0018)	0.0994
Mean velocity (MV), cm/s			
IVC	11.546 (10.590–12.501)	9.8446 (9.1688–10.520)	0.0045*
RFV	3.1701 (2.7576–3.5826)	3.6796 (3.4033–3.9560)	0.0435*
LFV	3.3659 (2.9615–3.7702)	3.9251 (3.5974–4.2528)	0.0345*
RGSV	1.1834 (0.8654–1.5015)	1.5070 (1.3000–1.7141)	0.0928
LGSV	1.1918 (0.8689–1.5147)	1.6853 (1.4044–1.9663)	0.0234*
RPV	1.3879 (1.1994–1.5764)	1.6687 (1.5223–1.8152)	0.0208*
LGV	−0.6674 (−1.6155 to 0.2806)	0.5074 (0.1117–0.9031)	0.0250*

* *P*<0.05

IVC, inferior vena cava; LEIV, left external iliac vein; LFV, left femoral vein; LG, left calf region; LGSV, left great saphenous vein; LGV, left gonadal vein (left ovarian vein); LPV, left popliteal vein; REIV, right external iliac vein; RFV, right femoral vein; RG, right calf region; RGSV, right great saphenous vein; ROI, region of interest; RPV, right popliteal vein.

The variables that most strongly differentiated the female patients with PCS from those non-PCS were SV in the great saphenous veins (right great saphenous vein, AUC=54%, 95% CI=47–62%; left great saphenous vein, AUC=54%, 95% CI=47–62%), SV in the right calf region (AUC=73%, 95% CI=67–78%), SV in the left calf region (AUC=75%, 95% CI=70–81%), MF in the left external iliac vein (AUC=51%, 95% CI=44–58%), MF in the great saphenous veins (right great saphenous vein, AUC=54%, 95% CI=47–62%; left great saphenous vein, AUC=55%, 95% CI=48–62%), MF in the right calf region (AUC=73%, 95% CI=67–79%), MF in the left calf region (AUC=75%, 95% CI=69–81%), SD in the great saphenous veins (right great saphenous vein, AUC=53%, 95% CI=46–60%; left great saphenous vein, AUC=54%, 95% CI=47–61%), and SD in the left gonadal vein (AUC=54%, 95% CI=45–63%). (Table 5).

**Table 5:** Patients with pelvic congestion syndrome (*n*=81) versus non-PCS (*n*=239) by each venous segments and calves’ regions: ROC discrimination test.

Parameters	OR (95% CI)	*P*	AUC (95% CI)	P*
SV: stroke volume				
IVC	1.056 (1.014–1.098)	0.0079	0.5977 (0.5307–0.6648)	0.0043
Right FV	0.692 (0.480–0.997)	0.0482	0.5669 (0.4961–0.6377)	0.0640
Left FV	0.702 (0.504–0.978)	0.0365	0.5752 (0.5058–0.6445)	0.0336
Right PV	0.569 (0.344–0.940)	0.0276	0.5821 (0.5143–0.6499)	0.0176
Left PV	0.567 (0.342–0.939)	0.0275	0.5762 (0.5082–0.6442)	0.0281
LGV	1.359 (1.114–1.657)	0.0024	0.7621 (0.7056–0.8186)	<0.0001*
Right calf region	10.55 (3.618–30.78)	<0.0001	0.7288 (0.6704–0.7873)	<0.0001*
Left calf region	8.512 (3.170–22.854)	<0.0001	0.7538 (0.6963–0.8112)	<0.0001*
MF				
IVC	1.039 (1.007–1.073)	0.0173	0.5990 (0.5330–0.6650)	0.0033
Right FV	0.730 (0.536–0.996)	0.0468	0.5709 (0.4999–0.6418)	0.0503
Left FV	0.734 (0.554–0.973)	0.0314	0.5792 (0.5101–0.6483)	0.0246
Right PV	0.611 (0.398–0.939)	0.0245	0.5861 (0.5190–0.6533)	0.0120
Left PV	0.607 (0.394–0.935)	0.0235	0.5784 (0.5111–0.6457)	0.0225
LGV	1.322 (1.121–1.558)	0.0009	0.7602 (0.7035–0.8169)	<0.0001*
Right calf region	8.284 (3.154–21.761)	<0.0001	0.7269 (0.6684–0.7853)	<0.0001*
Left calf region	5.818 (2.456–13.780)	<0.0001	0.7514 (0.6939–0.8089)	<0.0001*
SD				
IVC	1.083 (1.025–1.143)	0.0042	0.6271 (0.5619–0.6923)	0.0001*
Right EIV	0.975 (0.900–1.055)	0.5268	0.5639 (0.4933–0.6345)	0.0762
Right PV	0.719 (0.540–0.957)	0.0238	0.5909 (0.5239–0.6580)	0.0079
LGV	0.913 (0.839–0.993)	0.0328	0.5393 (0.4533–0.6253)	0.3700
Right calf region	>999.9 (0.049–999.9)	0.0642	0.5393 (0.4785–0.6000)	0.2051
Left calf region	>999.9 (38.41–999.9)	0.0302	0.5514 (0.4908–0.6121)	0.0966
MV				
IVC	1.076 (1.025–1.130)	0.0031	0.6239 (0.5581–0.6898)	0.0002*
Right EIV	0.982 (0.923–1.046)	0.5758	0.5707 (0.5011–0.6404)	0.0465
Left EIV	0.946 (0.877–1.020)	0.1477	0.5590 (0.4889–0.6291)	0.0988
Right FV	0.892 (0.777–1.023)	0.1016	0.5606 (0.4907–0.6304)	0.0891
Left FV	0.904 (0.800–1.022)	0.1075	0.5606 (0.4905–0.6306)	0.0901
Right PV	0.749 (0.587–0.954)	0.0195	0.5929 (0.5265–0.6593)	0.0061*
Left PV	0.880 (0.722–1.073)	0.2061	0.5615 (0.4936–0.6295)	0.0758
LGV	0.921 (0.858–0.989)	0.0234	0.5413 (0.4557–0.6269)	0.3440
Right calf region	>999.9 (1.217–999.9)	0.0490	0.5401 (0.4777–0.6026)	0.2078
Left calf region	>999.9 (2.742–999.9)	0.0427	0.5333 (0.4728–0.5938)	0.2811
Negative value (reflux) in left gonadal vein				
SD of LGV	0.913 (0.839–0.993)	0.0328	0.5393 (0.4533–0.6253)	0.3700
MV of LGV	0.921 (0.858–0.989)	0.0234	0.5413 (0.4557–0.6269)	0.3440

The radar chart visualized the discrimination ability of QFlow parameters in each venous segments between PCS and non-PCS (Fig. 4B).

### Verification of 2D PC-MRI hemodynamic analysis of left gonadal vein by angiogram (diagnostic golden standard of the gondal venous reflux.)

Fourteen patients underwent embolization of the left gonadal vein in accordance with the findings of preoperative 2D PC-MRI hemodynamic analysis and clinical symptoms. The data of these patients are listed in Table 6. We compared the findings of 2D PC MRI QFlow and the venogram during left gonadal vein embolization. The negative values of MV and presence of BFV in the left gonadal vein corresponded to the degree of venous reflux in the left gonadal vein during the venous intervention^30^.

**Table 6:** Patients with pelvic congestive syndrome receiving left ovarian venous (LOV) embolization.

No	Age	Comorbid	Procedure	VAS	SVP-S	SVP-V	SVP-P	BFV	MV	2DPC MRI LOV reflux	Angiogram documented LOV reflux
1	40	Nil	LOV embolization	7	S1,2,3a	V2,3a	Reflux	4.35	−7.72	Reflux	Grade 3
2	55	insomnia	LOV embolization	7	S2,3a	V2,3a	Reflux	4.54	−11.93	Reflux	Grade 3
3	45	Nil	LOV embolization; GSV truncal ablation; Foamy sclerotherapy	4	S2,3c	V2	Reflux	0.81	−4.87	Reflux	Grade 3
4	44	Nil	LOV embolization	5	S2	V2	Reflux	0.18	2.36	biphasic/stasis	Grade 2
5	45	Nil	LOV embolization	7	S1,2,3c	V2	Reflux	1.4	−3.85	Reflux	Grade 3
6	54	Nil	LOV embolization; GSV truncal ablation; Foamy sclerotherapy	3	S2,3c	V2,3ab	Reflux	4.13	−6.06	Reflux	Grade 3
7	45	Nil	LOV embolization	6	S2,3bc	V2,3b	Reflux	0.16	−1.22	Reflux	Grade 3
8	65	DM	LOV embolization; GSV truncal ablation; sclerotherapy	6	S2,3c	V2,3ab	Reflux	2.43	−7.1	Reflux	Grade 3
9	42	Nil	LOV embolization; GSV truncal ablation	4	S2,3c	V2	Reflux	0	3.52	Non-Reflux	Grade 1
10	42	Nil	LOV embolization	6	S2,3abc	V2,3a	Reflux	4.45	−7.28	Reflux	Grade 3
11	40	Nil	LOV embolization; GSV truncal ablation; Foamy sclerotherapy	4	S2,3bc	V2,3a	Reflux	0.29	2.15	biphasic/stasis	Grade 2
12	39	Nil	LOV embolization; GSV truncal ablation; Foamy sclerotherapy	5	S2,3a	V2,3a	Reflux	1.52	−4.08	Reflux	Grade 3
13	79	CAD; HTN	LOV embolization	7	S1,2,3a	V2,3ab	Reflux	4.12	−11.41	Reflux	Grade 3
14	62	Nil	LOV embolization; GSV truncal ablation	5	S2,3abc	V2,3ab	Reflux	2.91	−6.73	Reflux	Grade 3

The examination of this table shows the correlation coefficient matrix within the screening of BFV and MV toward Angio-LOV Reflux (golden standard). MV(QFlow) and Angio-LOV Reflux have the highly negative related, −0.7804, with *P*-value <0.05. BFV(QFlow) and Angio-LOV Reflux have the moderately positive relevant, 0.5869, with *P*-value <0.05. *Pearson product-moment correlation coefficient approach estimates the correlation and direction of any two variables.

Angio-LOV, angiogram documented left ovarian venous reflux; BFV, Backward flow volume; CAD, coronary artery disease; GSV, great saphenous vein; HTN, hypertension; LOV, left ovarian vein; MR-LOV reflux, 2DPC MR documented left ovarian vein direction; MV, mean velocity; SVP-P, pathology in symptoms/ varices/ pathophysiology classification; SVP-S, symptoms in symptoms/ varices/ pathophysiology classification; SVP-V, varices in symptoms/ varices/ pathophysiology classification; VAS, visual analog scale for pain.

## Discussion

Patients who come to our clinic for assistance with suspected pelvic and lower extremity venous disease commonly undergo NC-MRA for further diagnosis. This retrospective study aimed to examine the hemodynamic characteristics of patients diagnosed with pelvic congestion syndrome, which is more often observed in female patients than male patients. To ensure accurate observations, we excluded male patients from our study and grouped the remaining patients into those with pelvic congestion syndrome and those without. Furthermore, we included healthy volunteers without any venous disease in their pelvis and lower extremities as a control group to compare the findings between the two groups.

Since my team began routinely using noninvasive 2DPC-MRI (TRANCE MR) for the preoperative assessment of superficial venous interventions in 2018, we have become aware of the complex interactions within the pelvic venous system, including the gonadal, iliac, and even renal veins, which should be considered in the treatment of superficial venous diseases. Whiteley determined that approximately one in six (16.7%) women with leg varicose veins have them due to PCS^31^. In our study, a substantial proportion of patients of PCS frequently presented with venous claudication (26/81=32.1%) and underwent truncal ablation for leg symptoms (19/81=23.5%). The recognition and appropriate management of PCS remain uncertain due to of validated definitions, rigorous randomized clinical trials, and objective imaging criteria being lacking. In 2021, the American Vein and Lymphatic Society convened an International Working Group on Pelvic Venous Disorders to develop the SVP classification for PCS, which includes Symptoms (S), Varices (V), and Pathophysiology (P), with the pathophysiology domain encompassing the Anatomic (A), Hemodynamic (H), and Etiologic (E) aspects of the patient’s condition^3,28,32^. Varices (V) can be documented using US, which is not feasible for deep pelvic or retroperitoneal spaces in larger women^33^. CT and MRI with contrast media can delineate the anatomic relationship between the pelvic and retroperitoneal spaces; however, they require the injection of contrast agents and lack hemodynamic information^34^.

**Figure 5:**
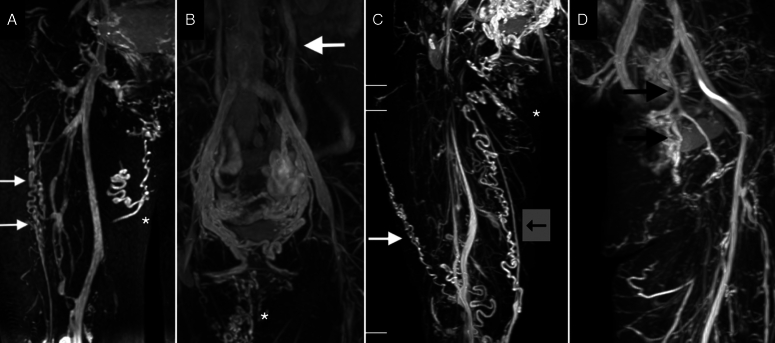
Typical morphologic figures in the patient with pelvic congestion syndrome (also present in the video). A. White arrow indicates the sciatic venous varices from the gluteal leak. White asteroid indicates the vulvar varices from the perineal leak. B. White arrow indicates the engorged left gonadal vein (left ovarian vein). White asteroid indicates the vulvar varices from the perineal leak. C. White arrow indicates the lateral thigh varices from the gluteal leak. Black arrow indicates the varices from inguinal leak. White asteroid indicates varices from the perineal leak. D. Black arrow indicates the obturator leak from pelvic veins.

In this study, we identified typical imaging features of PCS—including pelvic, gonadal, uterine, and extrapelvic varices—by using 2D PC-MRI. These varices—located in the vulva, perineum, and obturator regions—are challenging to visualize and quantify through US (Fig. 5). The 2D PC-MRI enhanced the precision of the SVP classification; it enables the objective identification of varicose locations, which patients can easily recognize, all without the need for radiation or contrast media. Moreover, the phase shift of pixels, or QFlow, can provide hemodynamic information on the pelvic veins and overall leg tissue. Caudal flow in the left gonadal vein was identified in almost half of the patients with PCS (39/81, 48.1%). Laparoscopic ligations and endovascular embolization of the gonadal veins have become effective therapeutic options for PCS^35–37^. With this hemodynamic information, physicians can approach venous interventions with greater confidence.

The QFlow pattern differed between the patients with PCS and the HCs. The patients with PCS exhibited higher SV, BFV, and MF in the calf region (interstitial pixel shift) and left gonadal vein segment than did the HCs. By contrast, the patients with PCS had lower SV, FFV, MF, SD, and MV in the great saphenous veins (Table 2). These findings suggest that the HCs had larger or faster saphenous venous drainage and less interstitial fluid flow than did the patients with PCS. The patients with PCS also had higher SV and MF in the calf region (interstitial pixel shift) and left gonadal vein segment than did the patients without PCS (Table 2).

**Figure 6:**
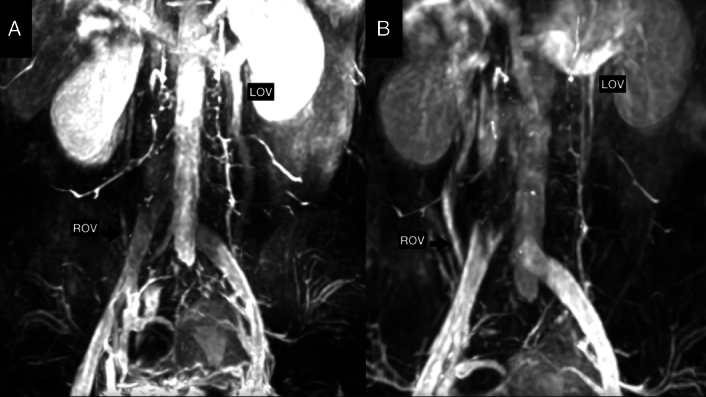
A female patient with pelvic congestion syndrome: Her chronic pelvic pain has a poor response to medication. A. Her engorged left ovarian vein was visualized (LOV). The caudal flow of the left ovarian vein was confirmed by quantitative 2D PC MRI. A small right ovarian vein could also be seen (black arrow). B. Her pelvic pain disappeared after left ovarian venous embolization. However, the pelvic symptoms recurred 3 years later. The second Quantitative 2D PC MRI showed the atrophic left ovarian vein (LOV), with a larger right ovarian vein (black arrow) than 3 years ago.

The Society for Vascular Surgery and American Venous Forum recommends endovascular embolization of gonadal veins to treat pelvic congestion with a 2B level of evidence^28^. However, the choice between unilateral or bilateral gonadal venous embolization remains a topic of debate^35,36,38^. Endovascular repair through iliac venous stenting may be beneficial in patients with pelvic venous insufficiency^1,39^. However, Gavrilov *et al*.^40^ reported that stenting of the left iliac vein alone (without ovarian vein embolization) relieved symptoms in only ~17% of patients with PCS and May–Thurner Syndrome. 2D PC-MRI (QFlow) accurately predicts reflux in the gonadal vein, as confirmed by a venogram during gonadal venous embolization, aiding the precise endovascular treatment of PCS. Moreover, it allows for the noninvasive re-evaluation of immediate outcomes and potential recurrence (Fig. 6). 2D PC MRI is especially suitable for use in young women who may be pregnant because it is nonradiation-toxic and does not require contrast agents^41^.

Vascular imaging requires careful consideration of the challenges presented by imaging patients in a supine position. The position is indeed an essential factor affecting venous flow. In addition, adjacent arteries or organs can easily compress venous structures, leading to conditions that are close to obstruction. NC-MRA using gated 3D TSE-STIR sequences is an innovative technique that acquires vein-only imaging, excluding arteries and background signals (e.g. soft tissue, bone, or fat). In our previous study, we noticed that after ultra-clear venous imaging became clinically available, many patients showed images of iliac vein compression even though they did not have any associated symptoms^14,42^. Additionally, we observed images of iliac vein compression in many healthy volunteers, which piqued our interest. To explore this issue further, we conducted two studies to explore the utility of NC-MRA combined quantitative 2D PC-MRI measurement performed in the supine position^23,43^. The two studies above demonstrate the usefulness of quantitative 2D PC-MRI measurements, which are associated with clinical symptoms and can improve the diagnostic ability of venous imaging with NC-MRA. In clinical practice, the ability to quantitatively assess venous flow provides a unique and valuable perspective that complements the static images provided by CT and MRI. For instance, the identification of retrograde flow or reduced venous return in the pelvic region can significantly influence treatment decisions, such as the need for embolization or other interventional procedures.

Quantitative 2D PC-MRI offers significant advantages. Primarily, it provides quantitative hemodynamic data, which is not typically available from standard CT or MRI. This quantitative aspect allows for more precise blood flow and velocity measurement, which is crucial for diagnosing and evaluating conditions like pelvic congestion syndrome. Moreover, our method reduces reliance on contrast agents and radiation, making it safer for repeated use, particularly in young women and potentially pregnant patients. Although there are doubts associated with supine imaging, the advantages and valuable insights obtained through quantitative 2D PC-MRI cannot be overstated, particularly in the realm of pelvic venous conditions. This technique is a major advancement in accurately and safely evaluating venous disorders with minimal risk to the patient.

The major limitations of this study include its nonrandomized design and the small sample. The control group was small, was younger in age, and included only women. In addition, there is not a comprehensive ultrasound venous hemodynamic report available in this study. A large sample size, longer follow-up periods for clinical correlation, and a randomized study design would be helpful for broader application in therapeutic protocols. Regarding the limitations of supine imaging, future research should focus on developing positional MRI techniques and protocols that simulate standing or sitting positions to replicate better physiological conditions affecting venous return.

In summary, this study explored the use of quantitative 2D PC-MRI for treating PCS. 2D PC-MRI provides an objective view of venous anatomy in patients with PCS, those without PCS, and HCs. The promising quantifications from QFlow analysis can aid in elucidating the pathogenesis of PCS and enhancing treatment outcomes.

## Ethical approval

This study was approved by the Institutional Review Board of Chang Gung Memorial Hospital (approval numbers: 201802137B0, 201901058B0, 202100938B0, and 202300005A3).

## Consent

Written informed consent was obtained from the patient for publication of this case report and accompanying images. A copy of the written consent is available for review by the Editor-in-Chief of this journal on request.

## Source of funding

National Scientific and technique council: NSTC 112-2314-B-182A-120-; NSTC 113-2314-B-182A-066 –; NSTC 112-2314-B-182A-120- and NSTC 113-2314-B-182A-083- and Chang Gung Memorial Foundation: CMRPG6M0421; CMRPG6N0091; NMRPG6N0101; CMRPG6P0251-3;. CMRPG6N0091, CMRPG6N0092, NMRPG6N0101, and NMRPG6P0091.

## Author contribution

C.-Y.L.: visualization, formal analysis, and data curation; C.-W.C.: writing the paper, study concept or design, and funding acquisition; C.-C.K.: data collection and funding acquisition; Y.-C.H.: visualization and software; C.-Y.L.: data collection and investigation; C.-C.L.: data collection; T.-Y.Y.: data collection, data analysis, or interpretation; S.-C.W.: methodology; S.-Y.C.: data collection; Y.-H.L.: methodology; M.Y.W.: data collection analysis and interpretation; C.-J.C.: visualization, formal analysis, data curation, and statistic consultation; Y.-K.H.: writing – original draft, writing – review and editing, and investigation.

## Conflicts of interest disclosure

The authors declare no conflict of interest.

## Research registration unique identifying number (UIN)

The study was registered as researchregistry10167.

## Guarantor

Yao-Kuang Huang.

## Data availability statement

The data is available upon reasonable request.

## Provenance and peer review

Not commissioned, externally peer-reviewed.

## Supplementary Material

SUPPLEMENTARY MATERIAL
